# Examining If Changes in the Type of School-Based Intramural Programs Affect Youth Physical Activity over Time: A Natural Experiment Evaluation

**DOI:** 10.3390/ijerph18052752

**Published:** 2021-03-09

**Authors:** Kathleen E. Burns, Ashok Chaurasia, Valerie Carson, Scott T. Leatherdale

**Affiliations:** 1 School of Public Health and Health Systems, University of Waterloo, Waterloo, ON N2L 3G1, Canada; a4chaurasia@uwaterloo.ca (A.C.); sleatherdale@uwaterloo.ca (S.T.L.); 2 Faculty of Kinesiology, Sport, and Recreation, University of Alberta, Edmonton, AB T6G 2R3, Canada; vlcarson@ualberta.ca

**Keywords:** intramurals, school physical activity, team intramurals, individual intramurals, adolescence, youth, students

## Abstract

(1) School-based physical activity programs such as intramurals provide youth with inclusive opportunities to be physically active, yet we know little about how types of intramurals (e.g., team and individual sports) may contribute to youth MVPA. This research aims to evaluate how real-world changes in types of intramurals available in schools impact youth physical activity over time. (2) This study used three years of longitudinal school- and student-level data from Ontario schools participating in year 5 (2016–2017), year 6 (2017–2018) and year 7 (2018–2019) of the COMPASS study. Data on types of intramural programs from 55 schools were obtained, baseline demographic characteristics were measured and data on physical activity and sport participation were collected on a sample of 4417 students. Hierarchical linear mixed regression models were used to estimate how changes in the type of intramurals associate with youth MVPA over time. (3) Regardless of participation, adding individual and team intramurals was significantly and positively associated with female MVPA in Y6. (4) The indirect, but positive relationship between adding individual and team intramurals and female MVPA may be explained by other characteristics of the school environment that are conducive to female MVPA.

## 1. Introduction

Moderate-to-vigorous physical activity (MVPA) is positively associated with physical and mental health among youth and is important in healthy development and disease prevention [1,2]. Despite these benefits, only one-third of Canadian youth are meeting the Canadian guidelines of at least 60 min of MVPA per day [3,4,5], and MVPA tends to decrease in youth as they age [6,7]. Physical inactivity is a major modifiable behavioural risk factor for premature mortality and lost life expectancy [8,9,10], and small increases in physical activity can have a positive impact on current health and the future risk of disease [11]. As a leading modifiable risk factor for disease and mortality, interventions targeting physical inactivity should be a public health priority to reduce the population-level health burden in future years.

Youth are an important target for physical activity interventions, because health behaviours (e.g., physical activity, sedentary behaviour and diet) continue to shape and form during this time [12,13,14,15]. As highlighted in the social ecological model, the environment is an important factor influencing health behaviour [16]. Of particular interest is the school-environment because school-level programs, policies and the built environment all interrelate to influence youth physical activity behaviour [17,18,19]. School-based physical activity programs can effectively target physical inactivity [20,21], especially if these programs are inclusive and accessible to a large proportion of the youth population [22]. Intramurals (e.g., intra-scholastic sports) are played or participated in by students within the same school, and are an example of such inclusive programs, as these activities typically do not involve a fee or a high level of skill to partake in [23].

Participation in intramurals is positively associated with youth physical activity [18,24,25,26], however there is limited knowledge on how these programs affect MVPA over time. Not only is the longitudinal effect of intramurals on youth MVPA not well understood, but there are different types of intramurals, including individual activities (e.g., yoga, running club) and team activities (e.g., soccer, tennis), which may differentially affect youth physical activity. Previous research suggests that individual and team sports have different effects on female and male sport participation, as female youth are more likely to participate in individual sports, while males are more likely to participate in team sports [27,28]. Additionally, participation in team sports is generally associated with more physical activity compared to individual sports [27,29], highlighting that types of intramurals may have differential effects on physical activity.

There is a paucity of literature available to help inform schools on which intramural options provide an effective impact on improving student physical activity levels. However, in real-world practice, schools continue to add and remove a variety of different intramural programs regardless of the lack of scientific evidence guiding those decisions. This creates a unique opportunity to generate practice-based evidence by evaluating the impact that these ongoing real-world changes to intramurals have on student physical activity within the context of a natural experiment [30]. As such, this research aims to use a natural experimental study design to evaluate how ongoing changes in school-level individual and team intramurals impact female and male youth MVPA over time. The results from this study will provide practice-based evidence that can be used by schools to help inform the offering of intramurals, to ultimately increase participation and physical activity among the youth population.

## 2. Materials and Methods

### 2.1. Study Design

The COMPASS study is a 9-year prospective cohort study (2012–2021) collecting hierarchical and longitudinal data from a convenience sample of secondary school students and the schools they attend in Alberta, British Columbia, Ontario and Quebec [31]. This study used COMPASS host study data from the 55 Ontario schools that participated in Year 5 (Y5 2016–2017), Year 6 (Y6 2017–2018) and Year 7 (Y7 2018–2019) of the COMPASS study. Schools in Alberta, British Columbia and Quebec were excluded from this study because of differences in provincial physical activity programs and policies. Ontario schools (*n* = 36) were excluded if they did not participate across all three years. The COMPASS study used active-information, passive-consent parent/guardian permission protocols, and active student assent, where students could refuse to participate at any time. Participating students completed the COMPASS student questionnaire (Cq), which is a paper-based, self-administered, anonymous survey, in class time annually. Senior administration at each participating school completed the School Policies and Practice (SPP) questionnaire online annually. Details on the COMPASS host study, including sampling and the Cq and SPP data collection tools, are available online (www.compass.uwaterloo.ca (accessed on 8 March 2021)). The COMPASS study was approved by the Human Research Ethics Board at the University of Waterloo (ORE 30118) and appropriate school board and school committees.

Only students who were in grade 9 (13–14 years old) and 10 (14–15 years old) at baseline (Y5) with linked data across all three years were included in the study. A total of 5514 students were linked for the three-year study period from the 55 Ontario schools. Main reasons for non-linkage were students transferring schools, students not providing data on grade in Y5 or Y6, students who were absent or had a spare period during the time of Y5 or Y6 data collection, those who left secondary school early, or inaccurate data provided to link measures on the Cq. Details on the methods of COMPASS data linkage are available elsewhere [32,33]. Only students with: (i) complete data on all covariates and (ii) complete data or monotone missingness on the outcome were included in the analysis for a final sample size of 4417 students.

This study utilized a longitudinal quasi-experimental study design, meaning that data on the outcome were measured at pre-intervention (Y5), intervention (Y6) and post-intervention (Y7) time points and were compared between non-randomized intervention and control groups [30]. This design is considered the gold standard research methodology in natural experimental studies, and important school- and student-level covariates were measured and controlled for through stratification and adjustment to mitigate bias due to confounding from lack of randomization [30].

### 2.2. Measures

#### 2.2.1. Outcome

Average daily MVPA was measured for students in Y5, Y6 and Y7 using the Cq. The Cq asks students to record their daily time (hours and/or minutes) spent engaging in hard and moderate physical activity each day for the last 7 days (e.g., Monday-Sunday) by the following two prompts: (1) “Mark how many minutes of HARD physical activity you did on each of the last 7 days. This includes physical activity during physical education class, lunch, after school, evenings, and spare time” and (2) “Mark how many minutes of MODERATE physical activity you did on each of the last 7 days. This includes physical activity during physical education class, lunch, after school, evenings, and spare time. Do not include time spent doing hard physical activities”. The Cq also provides students with descriptions of moderate and vigorous activities to aid with recall. Moderate physical activities are described as “physical activities include lower intensity activities such as walking, biking to school, and recreational swimming”, and vigorous physical activities are described as “physical activities include jogging, team sports, fast dancing, jump-rope, and any other physical activities that increase your heart rate and make you breath hard and sweat”. These descriptions align with definitions given by Canadian Society for Exercise Physiology (CSEP) that are used in physical activity research among youth [34]. Average daily MVPA is derived by summing the total time in minutes of moderate physical activity for each day (Monday–Sunday) and the total time in minutes of hard (vigorous) physical activity for each day (Monday–Sunday) and dividing this sum by 7 days. This self-reported measure of MVPA on the Cq has demonstrated satisfactory reliability and validity, making it an acceptable measure of MVPA in research involving school-age youth [35,36].

#### 2.2.2. School-Level Predictor: Type of Intramural Change

Type of intramural change was measured in the Ontario schools by comparing intramural data from year 5 (Y5) to year 6 (Y6). The offering of intramurals is distinctly separate from physical education and varsity sports. School administrators were asked in Y5 and Y6 to “Please select the intramural programs/club activities involving physical activity that were offered to students at your school during the past 12 months.” The intramural program selections include a variety of activities (e.g., soccer, basketball, yoga, running club) and include spaces for unlisted activities. School administrators were also asked to indicate whether the intramural offerings were for females only, males only, or co-ed. The intramurals were classified as team or individual sports based on the classifications found in Appendix A. Changes in team and individual intramurals from Y5 to Y6 were then determined by comparing the number of intramural programs offered between these years. Schools made many changes in team and individual intramurals from Y5 to Y6 (e.g., added and removed programs), and to ensure cell counts were large enough, these changes were categorized into five groups based on the quantity of types of intramural changes: (1) schools that primarily added individual intramurals from Y5 to Y6, (2) schools that primarily added team intramurals from Y5 to Y6, (3) schools that added the same number of individual and team intramurals from Y5 to Y6, (4) schools that added and removed the same number of individual and team intramurals from Y5 to Y6, and (5) schools that primarily removed team or individuals from Y5 to Y6 (reference). These groups were then coded into the following categories: (1) primarily added team, (2) primarily added individual, (3) added team and individual, (4) no net change and (5) removed intramurals (reference). Schools classified as: (4) no net change, are schools that removed an intramural for every intramural they added (e.g., added one team, removed one team and added on individual and removed one individual).

#### 2.2.3. Student-Level Correlates

Student-level sport participation and sociodemographic data that were known to associate with the predictor and/or outcome were measured by the Cq and included in the analyses. Data on intramural sport participation, varsity sport participation and community sport participation were measured at Y5, Y6 and Y7. *Intramural sport participation* is measured by asking “Do you participate in before-school, noon hour, or after school physical activities organized by your school? (e.g., intramurals, non-competitive clubs)”, followed by the response options of “No”, “Yes” and “None offered at my school”. Varsity sports are competitive sports played between students from different schools and typically involve a higher level of skill compared to intramurals. *Varsity sport participation* is measured by asking “Do you participate in competitive school sports teams that compete against other schools? (e.g., junior varsity or varsity sports)”, followed by the response options of “No”, “Yes” and “None offered at my school”. Community sports are competitive and non-competitive sports played by students outside of school. *Community sport participation* is measured by asking “Do you participate in league or team sports outside of school?”, followed by the response options of “No”, “Yes” and “None offered where I live”. Responses to all sport participation questions were recoded as “No (reference)” and “Yes” to ensure adequate cell counts. Demographic data were measured at baseline using the Cq and for the following variables with the corresponding response options in brackets: *gender* [female (ref), male], *ethnicity* [White, Black, Asian, Indigenous (First Nations, Métis, Inuit) Latin American/Hispanic, and Other)] and *weekly spending money* [0 CAD, 1 CAD to 5 CAD, 6 CAD to 10 CAD, 11 CAD to 20 CAD, 21 CAD to 40 CAD, 41 CAD to 100 CAD, more than 100 CAD, I do not know. *Ethnicity* and *weekly spending money* were collapsed into the following categories to ensure adequate cell count: *ethnicity* [white (ref), other], *weekly spending money* [Zero (ref), 1–20 CAD, 21–100 CAD and 100+ CAD].

#### 2.2.4. School-Level Correlates

School-level covariates that were known to associate with the predictor and/or outcome were measured and included in the analyses. The *number of intramural programs* and *school size* are both continuous variables measured at baseline using the SPP. Data on other school-level physical activity programs that may affect MVPA and/or intramural participation were obtained from the SPP and compared between Y5 and Y6 to derive the variable *changes in physical activity programs* which was categorized as either: “no changes in physical activity programs”, “added physical activity programs” or “removed physical activity programs”. *School neighbourhood median income* was obtained from the 2016 Canadian Census and are based on the median income of the area surrounding the school at baseline [37].

### 2.3. Analyses

All analyses were performed in SAS 9.4. Descriptive analyses were performed on school- (*n* = 55) and student-level characteristics (*n* = 4417). Chi-Square was used to examine exploratory differences between female and male students on student-level characteristics at baseline. An empty linear mixed regression model (i.e., intercept-only model) was used estimate the intraclass correlation (ICC) to determine the variability of MVPA between schools. Linear mixed models stratified by gender were used to estimate how changes in team and individual intramurals in Y5 to Y6 were associated with longitudinal MVPA. These models were hierarchical to account for clustering of students within schools and students over time and controlled for relevant student (grade, ethnicity, weekly spending money, intramural sport participation, varsity sport participation, community sport participation) and school (changes in physical activity programs, number of intramurals in Y5, school size, and school neighbourhood median income) factors. A novel modeling approach to program evaluation was employed to create an indicator variable representing the yearly change in type of intramurals: (i) type of intramural change in Y6 and (ii) type of intramural change in Y7. These indicator variables were included in the model and allowed for the assessment of their effect on MVPA at the intervention year (Y6) and post-intervention year (Y7). For Y7, the effect of type of intramural change was assessed under the assumption that changes from Y6 would continue into Y7. This novel modeling method has been utilized in previous research to assess changes in provincial policies on health outcomes [38], and to assess how changes in the environment affect alcohol use [39], tobacco and cannabis use [40] over time.

## 3. Results

### 3.1. School-Level Descriptive Statistics

The characteristics of the school-level sample are presented in Table 1. Specific to the change in types of intramurals from Y5 to Y6, 13 schools primarily added individual intramurals, 17 schools primarily added team intramurals, 5 schools added individual and team intramurals, 3 schools made no net change to intramurals and 17 schools removed intramurals. Five schools reported adding physical activity programs from Y5 to Y6 and no schools reported removing programs during this time. The mean school neighbourhood median income was 69,804 CAD (SD = 15,404 CAD) and the mean school size in Y5 was 669 students (SD = 288). Schools offered an average of 5.4 (SD = 4.1) intramural programs in Y5.

### 3.2. Student-Level Descriptive Statistics

The baseline demographic characteristics of the student-level sample are presented in Table 2 and the time-varying characteristics of the student-level sample are presented in Table 3. The purpose of these descriptive tables is to empirically observe the student-level categorical and continuous variables by presenting the frequency and percent for categorical variables and the mean and standard deviation for continuous variables. As shown in Table 2, 54% (*n* = 2402) of the sample were female, 73% (*n* = 3210) were white and $1-$20 was most frequently (43%, *n* = 1875) reported amount of weekly spending money. There were significant differences between males and females at baseline on weekly spending money (*p* < 0.0001). Referring to Table 3, intramural participation among female students was 38% in Y5, 36% in Y6 and 33% in Y7 and 39% in Y5, 37% in Y6 and 36% in Y7 among male students. Both female and male students participated in lower average daily MVPA over time, with females reporting 105 min (SD = 66) in Y5, 97 min (SD = 64) in Y6 and 89 min (SD = 61) in Y7 and males reporting an average of 117 min (SD = 68) in Y5, 109 min (SD = 68) in Y6 and 102 min (SD = 65) in Y7.

### 3.3. Results from Longitudinal Mixed Models

The ICC was calculated to estimate the amount of variation in MVPA that can be attributed to school-level differences for both female and male students. School-level differences accounted for 1.91% of the variability in MVPA among females and 2.09% among males, suggesting modest differences between schools on MVPA. Results from the linear mixed models are presented in Table 4. At baseline, female and male students in grade 10 reported significantly less average daily MVPA minutes compared to students in grade 9 (Females: $\hat{\beta }=\;$−8.383, *p* < 0.0001, Males:$\;\hat{\beta }=\;$−7.310, *p* = 0.001). Year was negatively associated with MVPA among females and males, although this relationship was only significant for females (females: $\hat{\beta }=\;$−7.226, *p* = 0.005, males $\hat{\beta }=\;$−1.070, *p* = 0.701). Participation in school and community sports were all positively associated with MVPA for both females and males. More specifically, students participating in intramurals (female: $\hat{\beta }=\;$5.040, *p* = 0.002, male: $\hat{\beta }=\;$9.722, *p* < 0.0001), varsity sports (female: $\hat{\beta }=\;$16.904, *p* < 0.0001, male: $\hat{\beta }=\;$18.045, *p* < 0.0001) and community sports (female: $\hat{\beta }=$ 26.105, *p* < 0.0001, male: $\hat{\beta }=\;$20.287, *p* < 0.0001) accumulated more daily MVPA on average compared to those who did not participate.

After controlling for intramural participation, adding individual and team intramurals was positively and significantly associated with female MVPA in Y6. In schools that added individual and team intramurals (*n* = 5), female students accumulated an average of 9.577 daily minutes of MVPA (*p* = 0.034) compared to female students attending schools that removed individual and team intramurals. The most frequent individual intramurals added among these five schools were rock climbing (*n* = 3), weight training (*n* = 2), yoga (*n* = 2) and outdoor club (*n* = 2). The most common team intramurals added among these five schools were dodgeball (*n* = 3), volleyball (*n* = 2) and soccer (*n* = 2). Primarily adding individual intramurals, primarily adding team intramurals, and no change in intramurals were all positively, but non-significantly associated with female MVPA in Y6. If these intramural changes in Y6 were maintained in Y7, primarily adding individual intramurals, primarily adding team intramurals and adding both individual and team intramurals were estimated to be positively, but non-significantly associated with MVPA in Y7. If schools that made no changes to individual and team intramurals in Y6 maintained this in Y7, it was estimated to have a negative, but non-significant effect on female MVPA.

Primarily adding individual and primarily adding team intramurals were both negatively, but non-significantly associated with male MVPA in Y6, after controlling for intramural participation. Adding individual and team intramurals and making no changes to intramurals were both positively and non-significantly associated with male MVPA in Y6, regardless of intramural participation. If these changes were to be maintained into Y7, primarily adding individual intramurals was estimated to be negatively and non-significantly associated with male MVPA, while primarily adding team intramurals, adding individual and team intramurals and making no changes to individual and team intramurals were all estimated to be positively, but non-significantly associated with male MVPA in Y7, independent of intramural participation.

## 4. Discussion

Using a large-linked sample of longitudinal data, we believe that this was the first study to evaluate how changes in the type of intramurals were associated with youth MVPA over time. We explored this association using an innovative new methodology that allowed us to examine how the effect of changes in type of intramurals affected MVPA in Y6 and into Y7 under the supposition these changes were maintained from Y6. Our results suggest that youth MVPA declines over time and adding team and individual intramurals positively impacts female MVPA in the year they were added, regardless of participation. Although other changes in type of intramurals did not provide a significant protective effect on this decline over time, these results nonetheless contribute to our understanding of how real-world changes in intramurals affect MVPA over time.

At baseline, youth in this sample achieved an average of 110 daily minutes of MVPA per day, which declined over time to 102 min in Y6 and 95 min in Y7. Additionally, female and male youth in grade 10 at baseline achieved significantly less daily average minutes of MVPA compared to youth in grade 9 at baseline. These findings are supported by other research, as younger youth typically accumulate significantly more daily MVPA minutes compared to older youth [4,41], and MVPA tends to decrease from grade 9 to grade 10 [42] and throughout high school [43]. MVPA declined over time for both male and female students, although the decline was only significant among female students. Decreases in youth physical activity over time have been well-documented [6,7,44,45,46,47,48] and may be attributed to decreases in sport participation [48,49], reduced physical education [50,51], and increased screen time and sedentary behaviour, [6,7,13,44] that are typical among youth during this time. Decreased sport participation in youth over time may be explained by many intrapersonal factors such as lack of enjoyment and lack of time [52,53]. Additionally, female youth generally have a larger decline in physical activity over time compared to males [54,55], which may explain why this relationship was only significant among females.

Intramural, varsity and community sport participation were all positively associated with MVPA among female and male youth. These findings are supported by previous research which suggests that intramural, varsity and community sport participation are all positively associated with youth MVPA [18,25,26,56,57,58]. Participation in community sports provided youth with more MVPA compared to intramural and varsity sports, which may be explained by the higher demand of community sports which typically includes frequent training and practice sessions in addition to competition [59]. It is important to note that intramurals and varsity are important opportunities for youth physical activity, especially for students who do not participate in community sports [60]. Additionally, community sports are less-inclusive than school-based intramural and varsity sports because they require transportation and are typically more time-consuming and more expensive compared to school-based sports [23]. Although community sports provided the most physical activity, all types of sport participation provided youth with daily MVPA, helping them achieve the recommended 60 min per day and providing other benefits associated with physical activity such as improved academic performance [61,62] and improved mental health [1,2].

The indictor variables included in the linear mixed models allowed for us to examine the associations between type of intramural change and MVPA in Y6 and Y7. After controlling for intramural participation, adding individual and team intramurals positively impacted female MVPA in the year they were implemented, but not if these changes were continued into Y7. This suggests adding individual and team intramurals is effective in the immediate year for females, but not over time, regardless of whether students participated in these intramurals or not. To keep youth involved over time, schools should consider ongoing engagement and in-school promotion of intramurals. This is not the first study to highlight that school-level intramurals indirectly and positively impact student MVPA. Other research has found that regardless of intramural participation, adolescents attending high schools with more intramurals engaged in more physical activity compared to those attending schools with less intramurals [24]. Perhaps schools that implemented team and individual intramurals have other characteristics of the school environment that positively associate with MVPA, especially among females. For example, these schools may provide students with access to equipment and facilities that support participation in non-competitive (e.g., aerobics, weight classes, yoga) and nonorganized physical activity (e.g., walking, running, rock climbing), both of which positively associate with physical activity among females in early high school [63]. Female students may have the opportunity to participate in these activities outside of intramurals if schools have implemented physical activity policies such as providing open access to equipment and facilities before-school, after-school and during lunch. Although the effect of the school environment on youth MVPA over time was not examined in this study, future research should explore how policies and facilities associate with youth MVPA especially among females, considering they consistently achieve less MVPA compared to their male counterparts.

The lack of other significant findings suggests that changes in types of intramurals are not associated with MVPA over time. Multifaceted physical activity programs are most effective at increasing physical activity among youth, so perhaps intramurals, though not effective on their own, they may be effective as part of a more comprehensive physical activity programs that include changes to school-level curriculum, policy and environment [21,64]. Although changes in types of intramurals were not associated with MVPA, it is important highlight that these changes may impact other beneficial outcomes students may receive from intramural participation. These include improved mental health [1,2], improved academic performance [61,62], reduced substance use [65] and more sport sampling [47,58,66,67,68,69,70,71]. Considering the multitude of benefits associated with youth intramural participation, future research should examine the effect of type of intramurals on intramural participation among youth.

Finally, it is important to note that intramurals are offered as female-only, male-only and co-ed, which impact intramural participation and MVPA. For example, gender offering of intramurals appears to be especially important for female students, as females attending schools that offered female-only intramurals were more likely to participate in intramurals compared to those attending schools without such programs [72]. Additionally, female-only intramurals may generate higher levels of physical activity among females compared to co-ed programs [18,24,25,26]. The gender-offering of intramurals was beyond the scope of this study, however a direction for future research could be to examine how gender-offering of intramurals is associated with intramural participation and MVPA over time.

### Limitations

Firstly, schools were recruited using convenience sampling, which may limit the generalizability of the results. However, the COMPASS study has a large sample size and utilizes as active-information, passive-consent protocol to encourage participation and honest response [73]. This recruitment method has been shown to limit self-selection and response biases and generate more robust results [31]. Secondly, we did not examine changes in the gender-offering (e.g., female-only, male-only and co-ed) of intramurals in our analysis. The gender-offering of intramural programs should be considered, as this may affect participation and physical activity, especially among females. Thirdly, because schools make many changes to the types of intramurals each year, the intervention groups may have been diluted (e.g., primarily added team, versus added team only), making the association between these changes and MVPA difficult to detect. Lastly, it is possible that this study was under-powered at the school-level to detect associations between changes in types of intramural programs and MVPA over time.

## 5. Conclusions

This study found that females attending schools that added individual and team intramurals achieved significantly more average MVPA per day the year intramurals were added, compared to females attending schools that removed programs, regardless of intramural participation. This suggests that there are school-level characteristics that are positively associated with female physical activity beyond intramurals, such as access to physical activity facilities or equipment. Although other associations between changes in types of intramurals and MVPA were not significant, intramurals can be an important aspect of comprehensive school-based physical activity strategies. Intramurals and varsity sport participation were all positively associated with youth physical activity and schools should consider offering a variety of intramural and varsity sports to maximize participation and physical activity among students. Sport participation in youth is positively associated with physical activity later in life [47,58,66], therefore, school-based sports present an important investment in youth health that should be a priority for stakeholders.

## Figures and Tables

**Table 1:** Descriptive Statistics for School-Level Characteristics for the sample (*n* = 55) from Year 5 and 6 (2016–2017) of the COMPASS Study.

Variable		Frequency	%
Changes in Types of Intramurals from Y5 to Y6	Primarily Added Individual	13	23.7
	Primarily Added Team	17	30.9
	Added Individual and Team	5	9.1
	No Change	3	5.5
	Removed Programs (Reference)	17	30.9
Changes in Other Physical Activity Programs from Y5 to Y6	No Change (Ref)	50	90.9
	Added Programs	5	9.1
	Removed Programs	0	0
Variable		Mean	Standard Deviation
School Neighbourhood Median Income in Y5		$69,804	$15,404 Min: $31,763 Max: $107,702
School Size In Y5		669	288 Min: 136 Max: 1550
Number of Intramurals Offered in Y5		5.4	4.1 Min: 0 Max: 14

**Table 2:** Descriptive Statistics for Baseline Student-Level Characteristics for the sample (*n* = 4417) from Year 5 (2016–2017) of the COMPASS study.

Variable		Total *n* = 4417	Female (Ref) *n* = 2402 (54%)	Male *n* = 2015 (46%)			
		Frequency (%)	Frequency (%)	Frequency (%)	DF	Chi-Square	*p*-Value
Grade	Grade 9 (Ref)	2434 (55.1)	1335 (55.6)	1099 (54.5)	1	1.431	0.232
	Grade 10	1983 (44.9)	1067 (44.4)	916 (45.5)			
Ethnicity	White (Ref)	3210 (72.7)	1744 (72.6)	1466 (72.8)	1	0.036	0.849
	Other	1207 (27.3)	658 (27.4)	549 (27.2)			
Weekly Spending Money	Zero (Ref)	1130 (25.6)	541 (22.5)	589 (29.2)	3	107.400	<0.0001
	$1–$20	1875 (42.5)	1062 (44.2)	813 (40.3)			
	$21–$100	1065 (24.1)	628 (26.1)	437 (21.7)			
	$100+	347 (7.9)	171 (7.1)	176 (8.7)			

Percent values may not equal 100 due to rounding.

**Table 3:** Descriptive Statistics for Time-Varying Student-Level Characteristics for the sample (*n* = 4417) from Year 5 (2016–2017), Y6 (2017–2018) and Y7 (2018–2019) of the COMPASS study.

Variable		Total *n* = 4417			Female (Ref) *n* = 2402			Male *n* = 2015		
		Year 5	Year 6	Year 7	Year 5	Year 6	Year 7	Year 5	Year 6	Year 7
		Frequency (%)	Frequency (%)	Frequency (%)	Frequency (%)	Frequency (%)	Frequency (%)	Frequency (%)	Frequency (%)	Frequency (%)
Intramurals	No (Ref)	2733 (61.9)	2813 (63.7)	2903 (65.7)	1494 (62.2)	1542 (64.2)	1616 (67.3)	1239 (61.5)	1271 (63.1)	1287 (63.9)
	Yes	1684 (38.1)	1604 (36.3)	1514 (34.3)	908 (37.8)	860 (35.8)	786 (32.7)	776 (38.5)	744 (36.9)	728 (36.1)
Varsity	No (Ref)	2568 (58.1)	2529 (57.3)	2688 (60.9)	1464 (60.9)	1447 (60.2)	1558 (64.9)	1104 (54.8)	1082 (53.7)	1130 (56.1)
	Yes	1849 (41.9)	1888 (42.7)	1729 (39.1)	938 (39.1)	955 (39.8)	844 (35.1)	911 (45.2)	933 (46.3)	885 (43.9)
Community	No (Ref)	2109 (47.7)	2394 (54.2)	2781 (63.0)	1218 (50.7)	1377 (57.3)	1599 (66.6)	891 (44.2)	1017 (50.5)	1182 (58.7)
	Yes	2308 (52.3)	2023 (45.8)	1636 (37.0)	1184 (49.3)	1025 (42.7)	803 (33.4)	1124 (55.8)	998 (49.5)	833 (41.3)
Variable		Mean (Standard Deviation)	Mean (Standard Deviation)	Mean (Standard Deviation)	Mean (Standard Deviation)	Mean (Standard Deviation)	Mean (Standard Deviation)	Mean (Standard Deviation)	Mean (Standard Deviation)	Mean (Standard Deviation)
MVPA (min/day) *		110 (67) *n* = 4417	102 (66) *n* = 4414	95 (64) *n* = 4375	105 (66) *n* = 2402	97 (64) *n* = 2400	89 (61) *n* = 2380	117 (68) *n* = 2015	109 (68) *n* = 2014	102 (65) *n* = 1995

MVPA = Moderate-to-Vigorous Physical Activity. * Note that the sample sizes for MVPA in Y6 and Y7 are different compared to baseline as well as Y6 and Y7 of other covariates. This is because some subjects included in the model are missing MVPA data in Y6 and Y7 (i.e., monotone pattern).

**Table 4:** Linear Mixed Models examining the association between changes in intramural programming in Y6 on MVPA in Y6 and Y7 of the COMPASS Study stratified by gender.

Variable		Female *n* = 2402			Male *n* = 2015		
		Estimate	95% CI	*p*-Value	Estimate	95% CI	*p*-Value
Effect of Type of Intramural Change on MVPA in Y6	Removed Intramurals (Reference)	—		—	—	—	—
	Primarily Added Individual	5.545	−1.054–12.144	0.100	−2.238	−9.884–5.407	0.566
	Primarily Added Team	2.929	−3.054–8.912	0.337	−3.172	−10.048–3.704	0.366
	Added Individual and Team	9.577	0.726–18.429	**0.034**	1.539	−8.320–11.397	0.760
	No Net Change	2.272	−4.010–8.553	0.478	5.149	−1.784–12.082	0.145
Effect of Type of Intramural Change on MVPA in Y7	Removed Intramurals (Reference)	—	—	—	—	—	—
	Primarily Added Individual	1.169	−5.469–7.807	0.730	−2.962	−10.60–4.680	0.448
	Primarily Added Team	4.874	−1.125–10.873	0.111	0.241	−6.650–7.132	0.945
	Added Individual and Team	1.527	−7.369–10.423	0.737	1.300	−8.667–11.267	0.798
	No Net Change	−1.441	−11.989–9.107	0.789	8.782	−2.793–20.357	0.137
Grade	Grade 9 (Ref)	—	—	—	—	—	—
	Grade 10	−8.383	−12.086–−4.681	**<0.0001**	−7.310	−11.434–−3.186	**0.001**
Year		−7.226	−12.207–−2.246	**0.005**	−1.070	−6.527–4.388	0.701
Intramural Sport Participation	No (Ref)	—	—	—	—	—	—
	Yes	5.040	1.806–8.274	**0.002**	9.722	6.044–13.401	**<0.0001**
Varsity Sport Participation	No (Ref)	—	—	—	—	—	—
	Yes	16.094	12.400–19.747	**<0.0001**	18.045	13.944–22.147	**<0.0001**
Community Sport Participation	No (Ref)	—	—	—	—	—	—
	Yes	26.105	22.804–29.405	**<0.0001**	20.287	16.496–24.078	**<0.0001**

Models controlled for changes in physical activity programs in Y6, median school neighbourhood income in Y5, school enrolment in Y5, number of intramurals in Y5, ethnicity and weekly spending money. Values significant at α= 0.05 are bolded.

## Data Availability

The datasets generated and analyzed for this study will not currently be shared because this is an ongoing study; however, access to the data supporting the findings of this study can be requested at https://uwaterloo.ca/compass-system/information-researchers (accessed on 8 March 2021).

## Appendix A

**Table A1:** Classification of intramurals into team and individual sports.

Team	Individual
Soccer	Yoga
Cheerleading	Dance
Ball Hockey	Outdoor Club
Badminton	Mountain Biking
Basketball	Skiing
Volleyball	Weight Training Club
Baseball/Softball	Rock Climbing
Dodgeball	Fitness Club (e.g., CrossFit, Zumba)
Ultimate Frisbee	Running Club
Hockey	Walking Club
Other: Rugby	Other: Swimming
Other: Football	Other: Archery
Other: Tennis	
